# Arrhythmogenic right ventricular cardiomyopathy/dysplasia

**DOI:** 10.1186/1750-1172-2-45

**Published:** 2007-11-14

**Authors:** Gaetano Thiene, Domenico Corrado, Cristina Basso

**Affiliations:** 1 Pathological Anatomy, Department of Medical-Diagnostic Sciences and Special Therapies, University of Padua Medical School, Padua, Italy

## Abstract

Arrhythmogenic right ventricular cardiomyopathy/dysplasia (ARVC/D) is a heart muscle disease clinically characterized by life-threatening ventricular arrhythmias. Its prevalence has been estimated to vary from 1:2,500 to 1:5,000. ARVC/D is a major cause of sudden death in the young and athletes. The pathology consists of a genetically determined dystrophy of the right ventricular myocardium with fibro-fatty replacement to such an extent that it leads to right ventricular aneurysms. The clinical picture may include: a subclinical phase without symptoms and with ventricular fibrillation being the first presentation; an electrical disorder with palpitations and syncope, due to tachyarrhythmias of right ventricular origin; right ventricular or biventricular pump failure, so severe as to require transplantation. The causative genes encode proteins of mechanical cell junctions (plakoglobin, plakophilin, desmoglein, desmocollin, desmoplakin) and account for intercalated disk remodeling. Familiar occurrence with an autosomal dominant pattern of inheritance and variable penetrance has been proven. Recessive variants associated with palmoplantar keratoderma and woolly hair have been also reported. Clinical diagnosis may be achieved by demonstrating functional and structural alterations of the right ventricle, depolarization and repolarization abnormalities, arrhythmias with the left bundle branch block morphology and fibro-fatty replacement through endomyocardial biopsy. Two dimensional echo, angiography and magnetic resonance are the imaging tools for visualizing structural-functional abnormalities. Electroanatomic mapping is able to detect areas of low voltage corresponding to myocardial atrophy with fibro-fatty replacement. The main differential diagnoses are idiopathic right ventricular outflow tract tachycardia, myocarditis, dialted cardiomyopathy and sarcoidosis. Only palliative therapy is available and consists of antiarrhythmic drugs, catheter ablation and implantable cardioverter defibrillator. Young age, family history of juvenile sudden death, QRS dispersion ≥ 40 ms, T-wave inversion, left ventricular involvement, ventricular tachycardia, syncope and previous cardiac arrest are the major risk factors for adverse prognosis. Preparticipation screening for sport eligibility has been proven to be effective in detecting asymptomatic patients and sport disqualification has been life-saving, substantially declining sudden death in young athletes.

## Diseases name and synonyms

Arrhythmogenic right ventricular cardiomyopathy/dysplasia

ARVC/D

## Background

### Definition

Arrhythmogenic right ventricular cardiomyopathy/dysplasia (ARVC/D) is a unique heart muscle disease, clinically characterized by non-ischemic ventricular arrhythmias originating from the right ventricle (RV), at risk of cardiac arrest. It is one of the major causes of sudden death in the young and in the athletes. The pathology consists of progressive dystrophy of the RV myocardium with fibro-fatty replacement.

In the last 25 years, it was possible to identify the disease [1], to realize its heredo-familiar character [2] and the risk of sudden death [3], to report the pathology [4], to put forward clinical diagnostic criteria [5], to find therapeutic measures [6] and, finally, to discover the genetic background [7].

### Epidemiology

The prevalence of approximately 1 in 5,000 people has been estimated [8]. The exact prevalence of ARVC/D, however, is unknown and could be higher than the estimated because of the existence of many non-diagnosed or misdiagnosed cases.

In the Veneto Region, Italy, the prevalence of the disease has been estimated to vary from 1:2,000 to 1:5,000 [2].

## Historical notes

The disease was first described by Giovanni Maria Lancisi in 1736, who in his book *De Motu Cordis et Aneurysmatibus *reported a family with disease recurrence in four generations: the affected members presented with palpitations, heart failure, dilation and aneurysms of the RV and sudden death [9].

Dalla Volta *et al*. in 1961 reported a patient with "auricularization" of the RV pressure curve, emphasizing the peculiar hemodynamic picture of this non-ischemic heart muscle disease with RV behaving like an atrium [10]. However, we had to wait until the 80's to find the first clinical and pathologic series of patients with ARVC/D reported by Drs Marcus, Nava and Thiene [1-3].

Marcus *et al*. in 1982 reported the disease in adults, first emphasizing the origin of arrhythmias from the RV and the histopathological substrate consisting of fibro-fatty replacement of the RV free wall, accounting for epsilon wave and ventricular arrhythmias of RV origin with left bundle branch block (LBBB) morphology [1].

Familiar occurrence with an autosomal dominant pattern of inheritance and variable penetrance was first proven by Nava *et al*. in 1987–1988 [2,11].

In 1988, Thiene *et al*. observed an impressive series of sudden deaths in the young (≤ 35 years), with pathology consisting of ARVC/D, mostly occurring during effort, and all characterized by inverted T-waves in the right precordial leads at electrocardiogram (ECG) and apparently benign ventricular arrhythmias of LBBB morphology [3]. They accounted for 20% of all sudden deaths in the young and for the first time it was acknowledged that ARVC/D is another important cause of sudden death in the young [12].

The diagnostic imaging was then implemented to visualize the RV, either non-invasively through echocardiogram [13] or invasively through angiography [14].

Signal averaged ECG proved to be a sensitive tool to detect delay in the electric impulse transmission in the RV myocardium [15]. Improvements in the diagnostic procedures led the proposal of diagnostic criteria, whether major or minor, based upon RV dysfunction or structural alterations at imaging, tissue characterization at biopsy, repolarization or depolarization abnormalities and arrhythmias at the ECG, and family history of sudden death [5].

The first gene locus (*ARVD1*) was found by Rampazzo *et al*. in 1994 at chromosome 14q23 [16]. The pathological profile was described in detail by Basso *et al*. in 1996, emphasizing the frequent left ventricular (LV) involvement and an inflammatory component [4].

In 1995, ARVC/D was included among cardiomyopathies in the revised World Health Organization (WHO) classification [17] and progressive cell death (apoptosis) in myocyte was proven [18,19].

The need of an International Registry of the disease was raised [20] and two research programs were implemented in both sides of the Atlantic Ocean [21,22].

Meanwhile, spontaneous occurrence of ARVC/D have been observed in cats [23] and dogs [24].

The first gene defect was discovered in the recessive variant of the disease (identified since 1985) from the Naxos island and consisting of a cardiocutaneous syndrome (ARVC/D, palmoplantar keratosis and woolly hair) [25]. A deletion was detected in the gene encoding plakoglobin, a cell junction protein [26].

Thereafter, other genes encoding cell junction proteins were found defective in the dominant, classical form of ARVC/D: desmoplakin [27], plakophilin-2 [28], desmoglein-2 [29], desmocollin-2 [30]. These mutations were found to account for intercalated disk remodeling at the ultrastructural level [31]. Other variants of the disease were explained by mutation of the ryanodyne 2 receptor [32] and transforming growth factor **β**3 genes [33].

The discovery of these gene mutations allowed preliminary genotype-phenotype correlations to be made [34-37].

The implantable cardioverter defibrillator (ICD) represented a major advance in therapy [38].

Finally, electroanatomic mapping proved to be a sensitive tool for identifying areas of fibro-fatty replacement with low amplitude electrical activity [39].

Study of ARVC/D in transgenic mice [40,41] models may help elucidate the pathogenesis of the disease and elaborate therapeutic strategies.

## Clinical features and natural history

The onset occurs usually after childhood, with palpitations and/or syncope.

**Figure 1:**
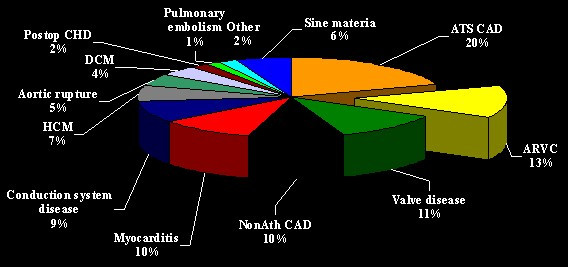
Graphic showing the various causes of juvenile sudden cardiac death in Northeast Italy. ARVC/D ranks second (13%) after atherosclerotic coronary artery disease. ARVC = arrhythmogenic right ventricular cardiomyopathy; ATS CAD = atherosclerotic coronary artery disease; DMC = dilated cardiomyopathy; HCM = hypertrophic cardiomyopathy; NonATS CAD = non-atherosclerotic coronary artery disease; Postop CHD = postoperative congenital heart disease.

**Figure 2:**
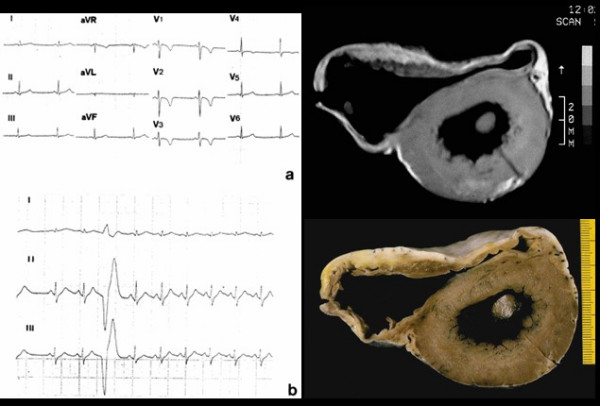
A 17 year old asymptomatic male athlete who died suddenly during a soccer game. 12 lead ECG showing inverted T waves up to V4 (a) and isolated premature ventricular beats (b). *In vitro *MRI (c) and corresponding cross section of the heart (d) show RV dilatation with anterior and posterior aneurysms.

**Figure 3:**
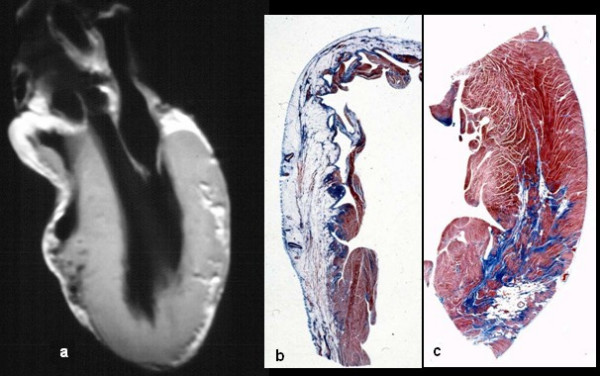
Same case of fig. 2. Note the biventricular involvement at long axis *in vitro *MRI (a), with transmural fibro-fatty replacement in the RV free wall (b) and focal subepicardial in the LV free wall (c).

The following clinical pictures of the disease have been observed [42]:

**1) Subclinical phase with concealed structural abnormalities**, during which the affected patient presents no symptoms, and cardiac arrest may be the first and last manifestation of the disease. ARVC/D has been reported as the second cause of sudden death in the young [3] (Figs. 1, 2, 3) and the main cause of sudden death in competitive athletes in the Veneto Region, Italy [43]. A subtle substrate exists, although does not yet manifest as an overt electrical disorder.

**Figure 4:**
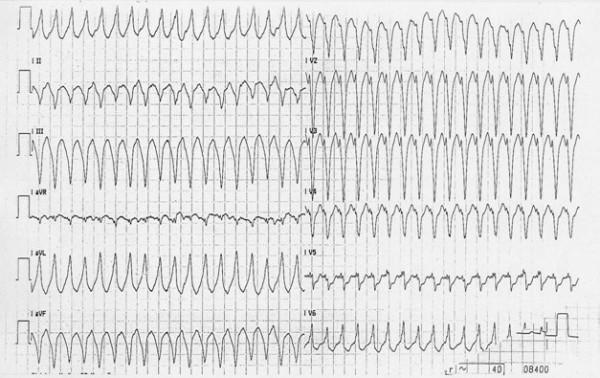
12 lead ECG recording of VT with left bundle branch block (LBBB) morphology.

**Figure 5:**
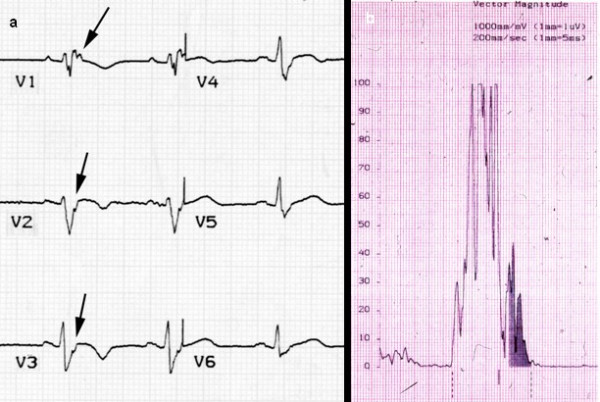
ECG recording: (a) post-excitation epsilon wave (arrows) in right precordial leads; (b) positive late potentials at signal-averaged electrocardiography (SAECG).

**2) Overt electrical disorder**, with palpitations and syncope. The most typical clinical presentation of the disease is symptomatic ventricular arrhythmias of RV origin, usually triggered by effort. Arrhythmias range from isolated premature ventricular beat to sustained ventricular tachycardia (VT) with LBBB morphology (Figs. 2, 4) up to ventricular fibrillation leading to cardiac arrest. The QRS morphology and axis during VT reflect its site of origin. A LBBB with inferior axis suggests an origin from the RV outflow tract (RVOT), while a LBBB with superior axis suggests an origin from the RV inferior wall. However, VTs with LBBB morphology are not specific for ARVC/D. Basal ECG may disclose inverted T waves in the right precordial leads (a T wave inverted beyond V1 after 14 years of age is almost pathognomonic of ARVC/D) [44] (Fig. 2). QRS enlargement of more than 110 ms and epsilon wave are strongly indicative of intraventricular impulse conduction delay [45]. Signal average ECG (wide amplitude superficial ECG) may help to disclose fragmented low amplitude late potentials at the end of the QRS complex [46] (Fig. 5). They correspond to the epsilon wave on surface ECG and reflect areas of slow intraventricular conduction due to islands of surviving myocardium interspersed with fatty and fibrous tissue, accounting for fragmentation of the electrical activation within the residual ventricular myocardium. VT/fibrillation may be easily triggered at the intracavitary electrophysiologic testing.

**3) RV failure. **The progressive loss of the RV myocardium may impair the mechanical function of the RV and account for severe pump failure.

**Figure 6:**
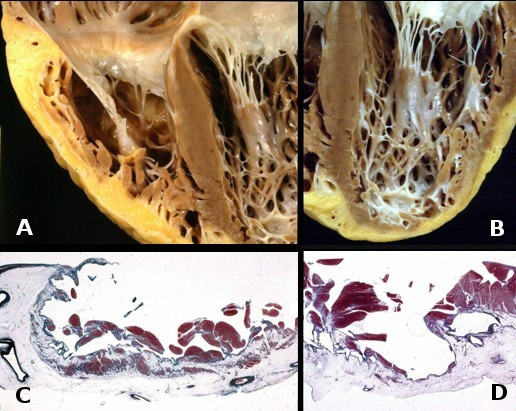
Heart specimen coming from heart transplantation. Note the biventricular involvement both at gross examination (a, b) and histology (c, d).

**4) Biventricular failure**. When the disease involves the ventricular septum and the LV, congestive heart failure occurs, mimicking dilated cardiomyopathy. Endocavitary mural thrombosis may occur, especially within aneurysms or in the atrial appendages when heart failure is complicated by atrial fibrillation, as to account for thromboembolism. In such conditions, contractile dysfunction may be so severe as to require cardiac transplantation (Fig. 6). Clearly, when the LV is affected, ventricular arrhythmias may appear polymorphic, originating from different cardiac regions. The occurrence of fatal outcome, mostly sudden death, varies between 0.1–3% per year in adults with diagnosed and treated ARVC/D, but it is unknown and may be higher in adolescents and young adults, in whom the disease is concealed and the first manifestation can be sudden death.

## Pathology and pathogenesis

**Figure 7:**
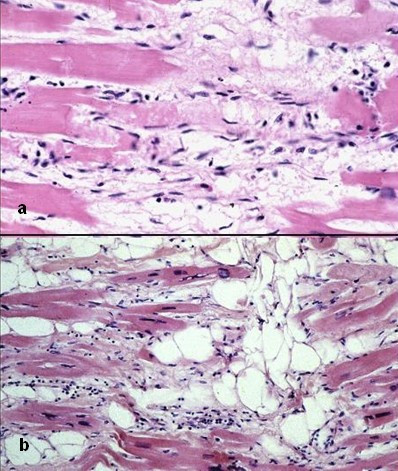
Typical histologic features of ARVC/D. Ongoing myocyte death (a) with early fibrosis and adipocytes infiltration (b).

The disease consists of a replacement of the myocardium of the RV by fibro-fatty tissue [3,4]. The phenomenon is progressive with time, starts from the epicardium and eventually extends down to reach the endocardium as to become transmural. This implies a weakness of the free wall resulting in RV dilatation and aneurysms, typically located at the inferior, apical and infundibular walls (the so-called triangle of dysplasia) [1]. The fibro-fatty replacement of the myocardium interferes with intraventricular conduction of the electric impulse accounting for delay (late potentials, epsilon wave, parietal right bundle branch block) and onset of re-entrant phenomena which are the mechanism of ventricular arrhythmias. Fatty infiltration of the RV has not to be considered "*per se*" a sufficient morphologic hallmark of ARVC/D. A certain amount of intramyocardial fat is present in the RV antero-lateral and apical region even in the normal heart and increases with age and body size. Moreover, ARVC/D should be kept distinct from adipositas cordis. Presence of replacement-type fibrosis and myocyte degenerative changes are essential to provide a clear-cut diagnosis, besides remarkable fat replacement [47] (Fig. 7).

The myocardial atrophy is progressive with time and by no way is present at birth, as seen in Uhl's disease, a congenital heart defect in which the RV myocardium failed to develop during embryonic life [48]. Instead, the myocardial loss is the consequence of cell death occurring after birth, usually during childhood [49]. An apoptotic mechanisms of myocyte death has been proven, either at post-mortem [18] or *in vivo *in endomyocardial biopsy specimens [19].

More than half of the hearts studied at post-mortem disclosed LV involvement, usually limited to the subepicardium of the postero-lateral free wall [50]. The involvement of the ventricular septum is rare, probably because it is not a subepicardial structure. In the most severe cases requiring transplantation, aneurysms may be seen also in the LV [51].

Histology of the RV myocardium discloses severe atrophy of the myocardium, replaced by fibro-fatty tissue, which should be regarded as an healing phenomenon following myocyte deaths [4]. Fibrous tissue, present in variable amounts, is an essential part of the healing process and plays a fundamental role in the intraventricular conduction delay of the electrical impulse, which is at the basis of onset of the life-threatening arrhythmias.

Death of single or multiple myocytes may be seen at histology, as proof of the acquired nature of myocardial atrophy, and may be associated with inflammatory infiltrates [52].

Myocardial inflammation may be seen in up to 75% of hearts at autopsy, and probably it plays a role in triggering ventricular tachyarrhythmias [53]. Nobody knows whether inflammation is a reactive phenomenon to cell death, or whether it is the consequence of an infection or immune mechanism. Viruses have been detected in the myocardium of some ARVC/D patients and have been claimed to support an infective etiology of the disease [54]. Others say that the viruses are innocent bystanders or that spontaneous cell degeneration may serve as a milieu favoring viral settlement in the myocardium [55].

**Figure 8:**
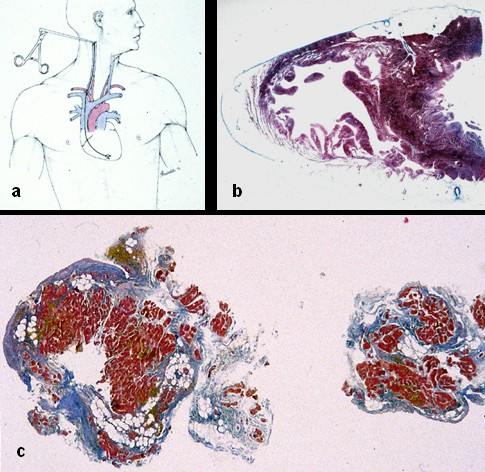
*In vivo *tissue characterization by endomyocardial biopsy: a) transvenous jugular approach of the bioptome; b) cross section of the heart showing that the septum is spared to underlie the need to perform the biopsy at the level of the RV free wall; c) fibro-fatty replacement of two bioptic samples.

Transvenous endomyocardial biopsy may be of great help for an *in vivo *morphological demonstration of fibro-fatty myocardial replacement [56] (Fig. 8). Samples should be retrieved from the RV free wall, since the fibro-fatty replacement is usually transmural and thus detectable from the endocardial approach and the ventricular septum is usually spared. A residual amount of myocardium <45%, due to fibrous or fibro-fatty replacement, has been proven to have a high diagnostic accuracy [57]. Histomorphometric criteria of endomyocardial biopsy from different sites of the RV are currently under evaluation.

## Clinical and differential diagnosis

*In vivo *diagnosis may be achieved by demonstrating alterations of the RV function and structure, typical depolarization and repolarization abnormalities, arrhythmias of the LBBB morphology, fibro-fatty replacement of the myocardium and existence of a family history.

Diagnostic criteria have been put forward [5] and divided into major and minor (Table 1). The diagnosis of ARVC/D would be fulfilled by the presence of two major, or one major plus two minor or four minor criteria from different groups.

**Table 1:** Criteria for diagnosis of ARVC/D

**1. Family history**	
***Major***	
Familial disease confirmed at necropsy or surgery.	
***Minor***	
Family history of premature sudden death (<35 years of age) due to suspected ARVC/D.	
Family history (clinical diagnosis based on present criteria).	
**2. ECG depolarization/conduction abnormalities**	
***Major***	
Epsilon waves or localized prolongation (>110 ms) of QRS complex in right precordial leads (V_1_-V_3_).	
***Minor***	
Late potentials on signal-averaged ECG.	
**3. ECG repolarization abnormalities**	
***Minor***	
Inverted T waves in right precordial leads (V_2 _and V_3_) in people >12 years of age and in absence of right bundle branch block.	
**4. Arrhythmias**	
***Minor***	
Sustained or nonsustained left bundle branch block-type ventricular tachycardia documented on ECG or Holter monitoring or during exercise testing.	
Frequent ventricular extrasystoles (>1000/24 h on Holter monitoring).	
5. Global or regional dysfunction and structural alterations*	
***Major***	
Severe dilatation and reduction of RV ejection fraction with no or mild LV involvement.	
Localized RV aneurysms (akinetic or dyskinetic areas with diastolic bulgings). Severe segmental dilatation of RV.	
***Minor***	
Mild global RV dilatation or ejection fraction reduction with normal LV.	
Mild segmental dilatation of RV.	
Regional RV hypokinesia.	
**6. Tissue characteristics of walls**	
***Major***	
Fibro-fatty replacement of myocardium on endomyocardial biopsy.	

* Detected by echocardiography, angiography, magnetic resonance imaging, or radionuclide scintigraphy.

Modified from McKenna *et al*. [5]

Morphologic changes accounting for global and/or regional dysfunction are detectable by echocardiography, angiography, cardiac magnetic resonance imaging (MRI), or radionuclide scintigraphy. Major criteria consist of severe dilatation and reduction in systolic function of the RV with no (or only mild) impairment of the LV; localized RV aneurysms (akinesia or diskinetic areas with diastolic bulgings); and severe segmental dilatation of the RV. Minor criteria are mild global RV dilatation and/or reduction in ejection fraction with normal LV, mild segmental dilatation of the RV free wall and regional RV hypokinesia.

**Figure 9:**
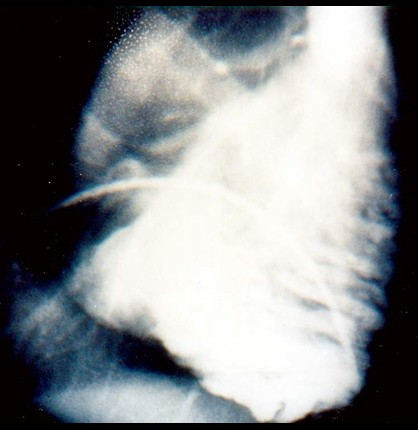
RV angiocardiography features of ARVC/D: RV dilatation with deep horizontal fissures in trabecular hypertrophy ("pile d'assiettes" profile) as well as subtricuspid aneurysm.

RV angiography is usually reported as the gold standard for the diagnosis of ARVC/D (Fig. 9). Angiographic evidence of akinetic/diskinetic bulgings localized in infundibular, apical and subtricuspid region has a high diagnostic specificity (>90%) [14].

**Figure 10:**
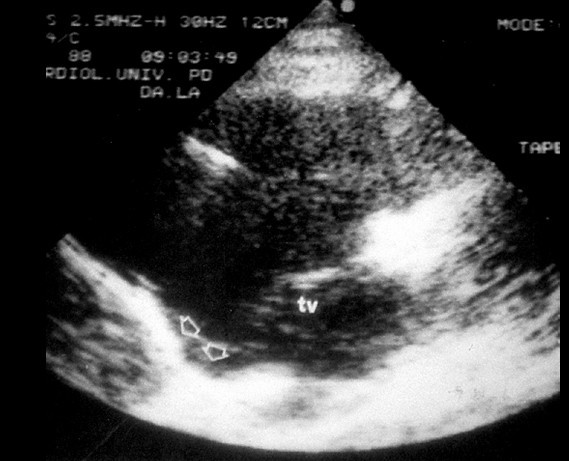
Two dimensional echocardiography findings in ARVC/D: note the presence of a typical inferior subtricuspid bulging (TV= tricuspid valve, parasternal long axis view of the RV).

Echocardiography is a non-invasive and widely used technique, and represents the first-line imaging approach for evaluating patients with suspected ARVC/D or for screening of family members (Fig. 10). Echocardiography also allows serial examinations aimed to assess the disease onset and progression during the follow-up of affected patients and family members. Both functional and structural abnormalities are detectable and, in the presence of typical echocardiographic features, contrast angiography or MRI may be avoided.

**Figure 11:**
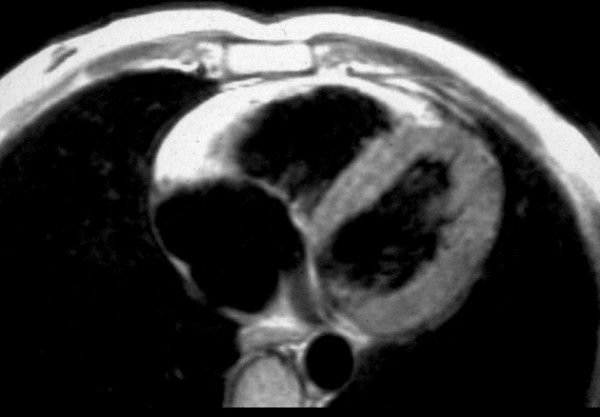
MRI in a patient affected by ARVC/D (long axis view of the right ventricle): note the transmural diffuse bright signal in the RV free wall on spin echo T1 (a) due to massive myocardial atrophy with fatty replacement (b).

MRI is an attractive imaging tool because it is non-invasive and has the ability to characterize tissue by distinguishing fat from muscle (Fig. 11). However, recent studies have shown a high degree of interobserved variability in assessing fatty deposition, which may be observed even in normal hearts. Cine-MRI may be of value in estimating RV volume and wall motion abnormalities with akinesia, dyskinesia and aneurysms.

Radionuclide angiography is also an accurate non-invasive imaging technique for detection of global RV dysfunction and regional wall motion abnormalities. Its diagnostic concordance with RV angiography is nearly 90% [58].

Tissue characterization of the RV free wall with fibro-fatty replacement of the myocardium, as demonstrated on endomyocardial biopsy (Fig. 8) or surgical resection, is considered a major criterion.

In contrast, repolarization abnormalities consisting of inverted T-waves in right precordial leads (V2 and V3) in the absence of RBBB, in individuals older than 12 years of age, are considered a minor criterion (Fig. 2).

As far as depolarization/conduction abnormalities, epsilon wave or localized prolongation of the QRS complex >110 ms in V1-V3 is a major criterion, whereas the presence of late potentials on signal averaged ECG has to be considered minor (Fig. 5).

Also arrhythmias, like sustained or non-sustained VT with LBBB morphology (Fig. 6), on basal ECG, Holter or exercise testing and frequent premature ventricular beats, >1000 over 24 hour Holter monitoring, are considered minor.

Finally, family history is a major criterion when familial disease is confirmed at necropsy or surgery, whereas it is minor in case of family history of premature sudden death (<35 years) or a family history of clinical diagnosis based on the present criteria.

A modification of Task Force Criteria for the diagnosis of ARVC/D has been proposed in case of family members for early detection of the disease [59]. In first degree relatives of a patient, confirmed to be affected by ARVC/D, the presence of right precordial T-wave inversion (V2 and V3), or late potentials on signal-averaged ECG, or VT with LBBB morphology, or mild functional or morphological changes of the RV on imaging, should be considered diagnostic for familial ARVC/D. In addition, the threshold of premature ventricular beats has been reduced from 1000 to 200 over 24 hour Holter monitoring to appear indicative of familial disease expression (Table 2).

**Table 2:** Proposed modification of Task Force criteria for the diagnosis of familial ARVC/D

**ARVC/D in First-Degree Relative Plus One of the Following:**	
1. ECG	T-wave inversion in right precordial leads (V_2 _and V_3_)
2. SAECG	Late potentials seen on signal-averaged ECG
3. Arrhythmia	LBBB type VT on ECG, Holter monitoring or during exercise testing
	Extrasystoles >200 over a 24-h period*
4. Structural or functional abnormality of the RV	Mild global RV dilatation and/or EF reduction with normal LV
	Mild segmental dilatation of the RV
	Regional RV hypokinesia

ECG = electrocardiogram; EF = ejection fraction; LBBB = left bundle branch block; RV = right ventricle; SAECG = signal-averaged electrocardiography; VT = ventricular tachycardia. *Previously >1,000/24-h period in Task Force criteria. Modified from Hamid *et al*. [59]

**Figure 12:**
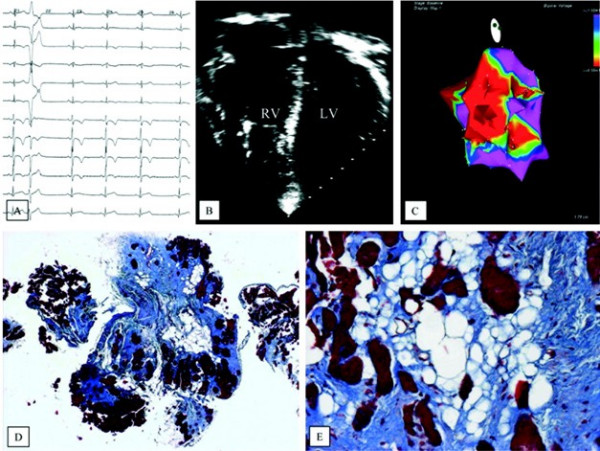
Invasive electro-anatomic mapping by CARTO. a) 12 lead ECG with inverted T waves up to V4 and LBBB premature ventricular beat; b) four chamber 2D echo showing RV dilatation and apical aneurysm; c) low voltages RV areas (red) by Carto mapping; d, e) extensive fibro-fatty replacement of the RV myocardium at endomyocardial biopsy (modified from Corrado *et al*., 2005) [39].

New tools for improving diagnostic accuracy have been introduced in recent years. Among non-invasive investigations, MRI with gadolinium late enhancement has been demonstrated to be able to detect fibrosis in the RV and LV myocardium [60]. Among invasive procedures, three dimensional electroanatomic mapping shows low-voltage areas which correspond to fibro-fatty myocardial replacement [39] (Fig. 12). It is able to differentiate ARVC/D from inflammatory cardiomyopathy mimicking ARVC/D, which shows a preserved electrogram voltage and has a better arrhythmic outcome. Moreover, this procedure is useful to distinguish early-minor forms of ARVC/D from idiopathic RVOT tachycardia, a non-familial benign arrhythmic disorder without substrate and a preserved electrogram voltage [61].

Of course, mutational analysis will help to establish with certainty who are the gene carriers, although asymptomatic. However, since these patients may not have the phenotypic expression of the disease, the Task Force criteria are critical to this assessment.

## Genetics

ARVC/D is heredo-familial in nearly 50% of cases, thus the ongoing myocardial atrophy may be genetically determined. The classical form is an autosomal dominant disease with variable penetrance [2,11]. In the 90's, gene loci have been mapped to various chromosomes, the first (ARVD1) by Rampazzo *et al*. to chromosome 14q23 [16]. The candidate genes were first searched for in those coding cytoskeleton or sarcomeric proteins, however ARVC/D revealed to be neither a cytoskeleton disease, like dilated cardiomyopathy, nor a sarcomeric disease, like hypertrophic cardiomyopathy. The key for interpretation came from a recessive form of ARVC/D, the so-called Naxos disease, a cardiocutaneous syndrome featured by palmoplantar keratosis, woolly hair and heart muscle disease [25,62]. Noteworthy, epidermic cells and myocytes share similar mechanical junctional apparatus, *i.e*. desmosomes and fascia adherens, which provides continuous cell-to-cell connection. This explains why genes coding proteins of the intercellular junction became candidate genes. Intercalated discs contain three types of cell-cell connection: gap junction (or nexus), adherens junction, and desmosome. Gap junctions mediate ion transfer between cells and each gap junction channel is a composite of two hemi-channels, or connexons, located within the cytoplasmic membrane of adjacent cells. The connexon, in turn, is formed by an assembly of six connexin subunits, of which connexin 43 (Cx 43) is the principal subtype in the human heart, but also connexins 40 and 45 are expressed at lower levels.

Synchronous contraction requires transmission of force between cells, which is accomplished *via *adheren junctions. The transmembrane component of an adherens junction, which establishes intercellular contact, is a cadherin, *i.e*. Ca^2+^-dependent glycoprotein. N-cadherin is the predominant isoform expressed in the human heart. Attached to the cytoplasmic tail of N-cadherin are **β**-catenin and plakoglobin (**γ**-catenin), both of which bind to **α**-catenin, which in turn, interacts directly with actin filaments within the sarcomere.

**Figure 13:**
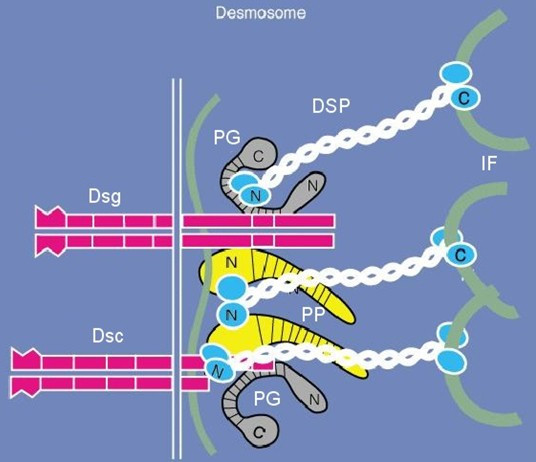
Scheme of the molecular structure of the desmosome, site of defective proteins in ARVC/D. PG = plakoglobin, DSP = desmoplakin, PP = plakophilin, DSG = desmoglein, DSC = desmocollin

Finally, desmosomes, together with adheren junctions, provide mechanical attachment between cells. However, in contrast to adheren junctions, desmosomes are not linked to the actin network, but with intermediate filaments, namely desmin in the heart and keratin in the skin. Proteins from three separate families assemble to form desmosomes: the desmosomal cadherins, armadillo proteins, and plakins (Fig. 13).

The genes encoding the desmosomal cadherins are clustered on chromosome 18q12.1 and four desmogleins (DSG1-4) and three desmocollins (DSC1-3) are recognized. The desmosomal cadherins comprise the transmembrane component of the desmosomal complex. Their extracellular domains interface directly with their counterparts on neighboring cells. Besides their role in cell adhesion, the desmosomal cadherins may function as regulators of morphogenesis. The intracellular portions of the desmosomal cadherins interact with proteins of the armadillo family, *i.e*. plakoglobin and plakophilin. Noteworthy, plakoglobin is also found in adhering junctions together with its homologue **β**-catenin. **β**-catenin, conversely, is not a constituent of desmosomes as it binds specifically to the classical cadherins. However, **β**-catenin has an additional nonadhesive function as a regulator of transcription, and a similar role has been postulated for plakoglobin. The plakophilins are found in the nucleus as well as the desmosome, although their function therein remains speculative. Binding sites for both plakoglobin and plakophilin are situated in the N-terminal domain of desmoplakin. At its C-terminal, desmoplakin anchors desmin intermediate filaments to the cardiomocyte surface [63].

A deletion in plakoglobin was first found in Naxos disease in 2000 [26], followed by mutation of demoplakin in 2002 [27], plakophilin-2 in 2004 [28], desmoglein-2 in 2006 [29] and desmocollin-2 also in 2006 [30] (Table 3). Thus, ARVC/D was found to be a cell junction disease also in the dominant form, with the plakophilin-2 as the most frequent disease gene [62-66]. Genotype-phenotype correlations revealed that the desmoplakin mutation is associated with a high occurrence of sudden death and frequent LV involvement [35], sometimes so pronounced as to deserve the term arrhythmogenic LV cardiomyopathy [67]. In contrast, the plakophilin mutation results in a more extensive disease manifestation with life-threatening ventricular arrhythmias [64]. Plakoglobin and plakophilin mutations leads to similar cardiac phenotypes with RV preponderance [68].

**Table 3:** Genes involved in ARVC

Abbreviation	Disease gene	Mode of transmission	Author, year [References]
JUP	Plakoglobin	AR	McKoy *et al*., 2000 [26]
RYR2	Cardiac Ryanodine receptor	AD	Tiso *et al*., 2001 [32]
DSP	Desmoplakin	AD	Rampazzo *et al*., 2002 [27]
PKP2	Plakophilin 2	AD	Gerull *et al*., 2004 [28]
TGFβ3	Transforming Growth Factor Beta-3	AD	Beffagna *et al*., 2004 [33]
DSG2	Desmoglein 2	AD	Pilichou *et al*., 2006 [29]
DSC2	Desmocollin 2	AD	Syrris *et al*., 2006 [30]

**Figure 14:**
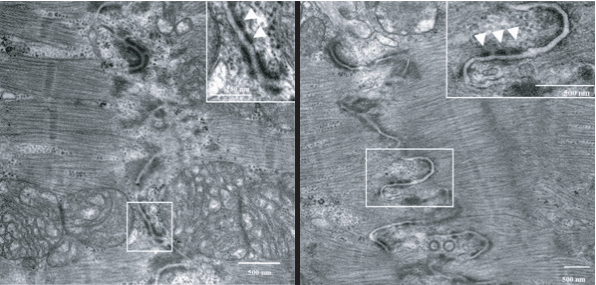
Transmission electron microscopy of the intercellular junction between two adjacent myocytes in ARVC/D. Note the presence of abnormal desmosomes, either long (arrows) or short-repeated structures (insert) (modified from Basso *et al*., 2006) [31].

Ultrastructural investigation in endomyocardial biopsy of patients with ARVC/D and cell junction gene mutations revealed intercalated disk remodeling with a decrease in the number and length of desmosomes and intercellular gap widening [31] (Fig. 14).

Cell junction protein mutations may account for a final common pathway, namely disruption of intercellular junction, myocyte death and structural changes, which are the substrate of life-threatening ventricular arrhythmias [69].

A recessive mutation of desmoplakin has been proven to explain another cardiocutaneous syndrome, *i.e*. Carvajal disease [70], characterized by keratoderma, woolly hair and a biventricular form of ARVC/D [71], with distinct ultrastructural abnormalities of intercalated discs and decreased immunoreactive signals for desmoplakin, plakoglobin and Cx 43.

Moreover, remodeling of intercalated disc may lead to widening of myocyte gap junction, which may also contribute to the arrhythmogenicity of the disease and enhance the risk of sudden death [72].

Two other gene defects have been reported to explain the disease so far. One is the gene encoding for the cardiac ryanodine receptor 2, which is located in the smooth sarcoplasmic reticulum and mediates calcium release for electroanatomical coupling (ARVD2 with polymorphic ventricular arrhythmias) [32]. Similar mutations have been shown to account for cathecolaminergic VT, a peculiar malignant arrhythmic disease in normal hearts [73]. Mild pathologic substrates have been described in ARVD2 [74], but clearly this disease is different from the classical form of ARVC/D and most probably we are dealing with the same nosographic entity as cathecolaminergic VT.

Another form of ARVC/D was found to be associated with regulatory mutations in the TGF**β **gene [33]. The gene defect may account for increased propensity for extracellular matrix production and adipogenesis. However, the report has been anedoctical and needs to be confirmed.

With gene mutations available, transgenic mice are now being developed to gain an insight into the etiopathogenetic mechanisms of the disease, with possible therapeutic implications [40,41].

## Risk stratification and therapy

Young age, "malignant" family history, QRS dispersion ≥ 40 ms, T-wave inversion beyond V1, LV involvement, VT, syncope or previous cardiac arrest are considered the major determinants for adverse prognosis and impending sudden death [75].

**Figure 15:**
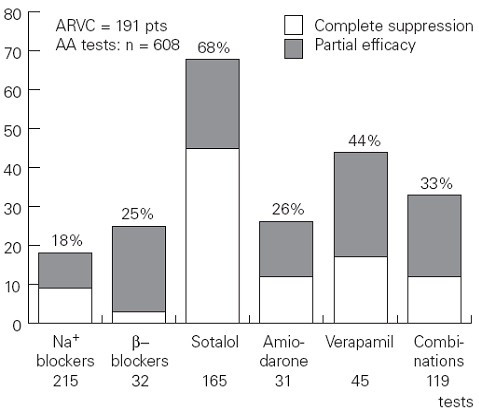
Efficacy rates of different antiarrhythmic drugs for treatment of ventricular tachycardia in ARVC/D (modified from Wichter *et al*., 2005) [76].

Different antiarrhythmic drugs have been employed: sodium blockers, **β**-blockers, sotalol, amiodarone, verapamil alone or combinations. Wichter *et al*. reported the various efficacy rates by demonstrating that sotalol is superior with a complete or partial efficacy in 68% of patients *vs *26% for amiodarone [6] (Fig. 15).

**Figure 16:**
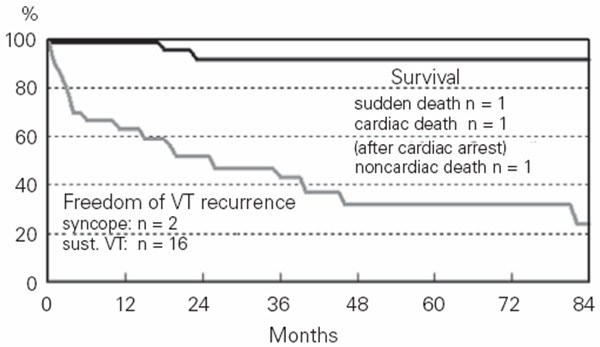
Long term follow-up after catheter ablation in ARVC/D (modified from Wichter *et al*., 2005) [77].

Catheter ablation has been accomplished in VT refractory to drug treatment [76]. Although the treatment may be effective in the short term, the procedure is associated with high rate of recurrence (40% freedom from recurrence at 3 years), clearly indicating its palliative nature. Nonetheless, in terms of survival, the outcome is quite good (Fig. 16).

**Figure 17:**
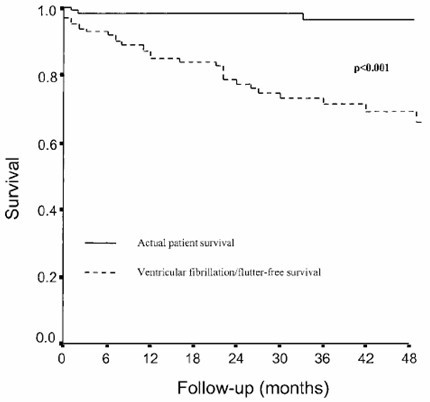
Long term follow-up after ICD implantation in ARVC/D patients for secondary prevention (modified from Corrado *et al*., 2003) [38].

ICD has been proven to be life-saving. Corrado *et al*. [38] found a freedom from electric shock, delivered in case of ventricular flutter/fibrillation, in 76% of patients at 48 months after implantation, whereas the survival curve was excellent with 96% of patients alive at the same time period. Considering that each episode followed by electric shock would have been fatal, 20% of patients were saved by ICD (Fig. 17).

In refractory congestive heart failure, cardiac transplantation is the only therapeutic option [50].

**Figure 18:**
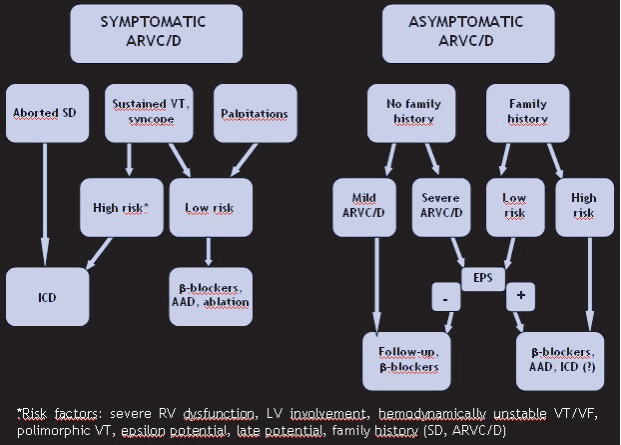
Proposed algorithm for management of ARVC/D (modified from Wichter *et al*., 2005) [76].

An algorithm for management of ARVC/D has been proposed (Fig. 18). In symptomatic patients, if an aborted sudden death occurred, ICD is imperative. In case of sustained VT and/or syncope, ICD is also indicated in the presence of risk factors (extensive RV dysfunction, LV involvement, polymorphic VT, late potentials and epsilon wave, family history) [77]. If sustained VT as well as palpitations occurred in low risk patients (none of the previous risk factors), antiarrhythmic therapy and/or ablation are indicated. Syncope is reported as a distinct high risk factor, particularly in the young [36,75].

In asymptomatic patients without a family history and a mild form of ARVC/D, **β**-blockers are recommended with follow-up control. If the form of ARVC/D is severe, electrophysiology-intracavitary testing is recommended. If negative, **β**-blockers and serial follow-up should be undertaken. If positive, ICD should be considered, as well as **β**-blockers and other antiarrhythmic drugs.

In the absence of symptoms and a family history, it is controversial whether electrophysiologic testing should be carried out even in patients at low risk. Finally, it should be underlined that, at present, no curative therapy has been postulated and clearly the aforementioned treatments are palliative. Gene therapy is still far from being established [78] and no treatment to limit disease progression has been conceived so far. Some drug interventions targeting the cascade of events leading to apoptosis and cell death, such as anticaspase agents, might be hypothesized. Corticosteroid treatment may be considered for myocardial inflammation, which is so frequently observed and probably aggravates the arrhythmogenicity: it is a hypothesis that needs to be investigated.

## Prevention of sudden death

**Figure 19:**
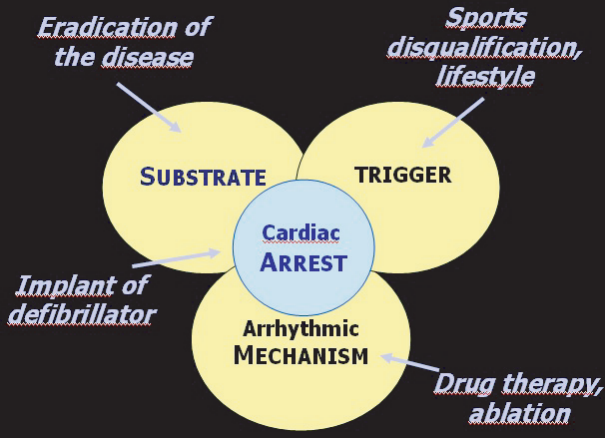
Diagram illustrating the different levels for prevention of sudden death in ARVC/D.

Cardiac arrest in ARVC/D is the consequence of a combination of various factors (substrate, trigger, arrhythmias) and measures for prevention should focus on these various steps (Fig. 19).

ICD aims to convert ventricular flutter/fibrillation into sinus rhythm for resuscitation from cardiac arrest. The device may be implanted in selected patients at risk or may be external, used on the spot in case of sudden cardiac arrest occurring in public sites, like sports courts, airports, schools, *etc*. The availability of this tool even at home for families at risk, should be considered, provided it is accompanied by a life-support training.

Drug therapy and ablation plays a fundamental role in the arrhythmic mechanism to prevent onset of life-threatening arrhythmias. The efficacy is, however, limited and the recurrence of arrhythmias quite high.

**Figure 20:**
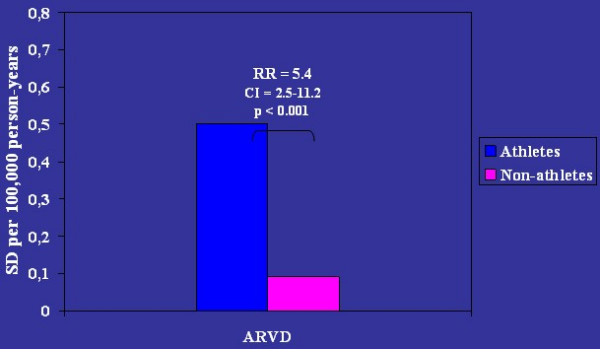
Relative risk of sport-related sudden death in ARVC/D (modified from Corrado *et al*., 2003) [79].

**Figure 21:**
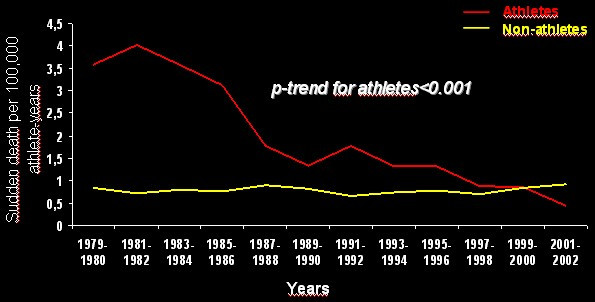
Trends of sudden cardiac death incidence in athletes *vs *non-athletes, Veneto Region of Italy, 1979–2002: note the sharp decrease (modified from Corrado *et al*., 2006) [80].

Effort, by volume overload and stretching of the RV myocardium, is considered a major trigger. Sport activity increases 5 fold the risk of sudden death in the young [79] (Fig. 20). Thus, identification of the asymptomatic ARVC/D carriers is crucial to avoid effort. Preparticipation screening and sport disqualification, with a choice of life style without strenuous efforts, has been shown to be quite effective in preventing sudden death in athletes [80]. In the Veneto Region, following implementation of obligatory preparticipation screening there was a sharp decline in sudden death in athletes from 1:28,000 per year in the pre-screening period to 1/250,000/year in the late screening period, mostly due to identification and disqualification of patients affected by ARVC/D [80] (Fig. 21).

A different life style may be safe "*per se*", regardless of the need of antiarrhythmic/ablation therapy or ICD. These are palliative, empiric treatments.

Curative therapy of the disease, the radical form of prevention of sudden death, may be accomplished in various ways:

a) Heart replacement, in case of refractory congestive heart failure and/or arrhythmias, with cardiac transplantation.

b) Some therapy to prevent myocyte death and inflammation, to block onset and progression of the disease at the pathobiological level. Nothing is available so far and transgenic animal models are ideal to investigate the pathogenesis of the diseases and to figure out curative therapies [40,41].

c) Repair of the defective genes at somatic level (gene therapy), a controversial and yet inconclusive approach.

d) Genetic counseling and birth control.

It must be underscored that the phenotypic expression of the gene defect, with the exception of cardiocutaneous syndromes, is only at the cardiac level and nowadays a series of effective measures are available to ensure normal life, with very low risk of premature death in affected patients.
